# Children with ADHD and EEG abnormalities at baseline assessment, risk of epileptic seizures and maintenance on methylphenidate three years later

**DOI:** 10.1186/s12991-024-00510-4

**Published:** 2024-06-21

**Authors:** Dobrinko Socanski, Geir Ogrim, Nezla Duric

**Affiliations:** 1 Department of Child and Adolescent Psychiatry, Østfold Hospital Trust, Fredrikstad, Norway; 2 Department of Child and Adolescent Psychiatry, Stavanger University Hospital, Stavanger, Norway; 3 Neuropsychiatric Team, Åsebråten Clinic, Østfold Hospital Trust, Fredrikstad, Norway; 4 Gillberg Neuropsychiatry Centre, Institute of Neuroscience and Physiology, University of Gothenburg, Gothenburg, Sweden; 5 Department of Child and Adolescent Psychiatry, Fonna Health Trust, Haugesund, Norway

## Abstract

**Purpose:**

This study aimed to assess the incidence of EEG abnormalities (EEG-ab) in children diagnosed with ADHD, investigate the risk of epileptic seizures (SZ) and maintenance on methylphenidate (MPH) over a three-year period.

**Methods:**

A total of 517 ADHD children aged 6–14 years were included. Baseline assessments included the identification of EEG-ab, ADHD inattentive subtype (ADHD-I), comorbid epilepsy, the use of antiepileptic drugs (AEDs) and the use of MPH. At the 3-year follow-up, assessments included the presence of EEG-ab, maintenance on MPH, AED usage, SZ risk in cases with EEG-epileptiform abnormalities (EEG-epi-ab), compared with control ADHD cases without EEG-epi-ab matched for age and gender.

**Results:**

EEG-ab were identified in 273 (52.8%) cases. No statistically significant differences were observed between the EEG-ab and EEG-non-ab groups in terms of age, gender, ADHD-I type or initial use of MPH. EEG non-epileptiform abnormalities (EEG-non-epi-ab) were found in 234 out of 478 (49%) cases without EEG-epi-ab. Notably, EEG-non-epi-ab occurred more frequently in the group of 39 cases with EEG-epi-ab (30/39 (76.9%) vs. 9/39, (21.3%), a subset selected for 3-year follow-up. At 3-year-follow-up no statistically significant difference was found in maintenance on MPH in ADHD cases with and without EEG-epi-ab. Nobody of ADHD cases without comorbid epilepsy or with comorbid epilepsy with achieved SZ freedom developed new SZ. Only 3 children with drug resistant epilepsy experienced SZs, without increase in SZ frequency. The disappearance rate of EEG-epi-ab was higher than that EEG-non-epi-ab (71.8% vs. 33.3%).

**Conclusions:**

Children with and without EEG-ab exhibited similar patterns of MPH use (initial use, positive response, and maintenance on MPH). The presence of comorbid epilepsy and EEG-ab, with or without EEG-epi-ab, was not associated with an increased risk of SZ despite the use of MPH.

## Introduction

Attention-deficit/hyperactivity disorder (ADHD) is a prevalent condition in children characterised by an inappropriate pattern of attention and/or hyperactivity-impulsivity. Key symptoms typically manifest before the age of 7 (DSM IV; ICD-10) or up to age 16 (DSM 5), with observable altered behaviour in at least two distinct settings [1–3].

There exists a notable interconnection between ADHD, epilepsy and abnormal EEG findings (EEG-ab). ADHD frequently coexists with epilepsy in children [4–9] where in children diagnosed with ADHD may also exhibit epilepsy and/or EEG abnormalities (EEG-ab). These abnormalities encompass both epileptiform abnormalities (EEG-epi-ab) and non-epileptiform abnormalities (EEG-non-epi-ab), such as slowing and/or irregularity of the background rhythm [10–13]. EEG-ab, particularly EEG-epi-ab, is prevalent in children with epilepsy [14]. Furthermore, EEG-ab is more frequently identified in children with ADHD [15] compared to their healthy counterparts, although these findings have not been thoroughly investigated. Earlier studies have reported increased theta activity in frontal-centra (F-C) area, and diminished alpha activity in children with ADHD. A pioneering EEG study conducted eight decades ago detected EEG-ab (heightened slow activity) in 59% (42/71) of children with behavioural disorders [16]. It has been posited that abnormal EEG in ADHD may signify an undefined developmental deviation [17, 18]. Quantitative EGG (Q-EEG), utilised in resting conditions or in combination with task conditions such as continuous performance tests (CPTs), has been instrumental in ADHD assessment and model development [19, 20]. Children with ADHD often display an excess of slow brain waves (theta) and an increased theta-to-beta (θ/β) power ratio during rest compared to non-ADHD controls [21–27].

However, comprehensive evidence is lacking regarding the frequency of EEG-ab, encompassing both EEG-non-epi-ab and EEG-epi-ab, recorded on standard awake EEG in children with ADHD. Moreover, knowledge gaps persist regarding the impact of EEG-ab on ADHD subtypes, the utilisation of methylphenidate (MPH) treatment and the risk of epileptic seizures (SZ) during long-term follow-up.

### Purpose

This study aims to explore the occurrence of EEG-ab on standard awake EEG recordings during ADHD assessments and elucidate their associations with age, gender, the prevalence of the ADHD inattentive subtype (ADHD-I), the use of MPH treatment, the risk of SZ and the maintenance on MPH three years post assessment.

## Materials and methods

### Participants and procedure

A total of 517 children diagnosed with ADHD (82.4% male), aged between 5 and 14 years, mean age 9.4 (+/-2.5) were included in the study. These participants were diagnosed at Stavanger University Hospital over a 6-year period, from January 2000 to December 2005. The diagnosis adhered to DMS-IV-TR criteria [28]. A retrospective chart review was conducted for all ADHD diagnoses during this period, and patients were prospectively collected and analysed at a single regional centre. During the study period, all children suspected of having ADHD underwent a routine awake EEG. A digitised 20-minute routine awake EEG with 21 electrodes (10–20 system), inclusive of hyperventilation and photic stimulation was administered to the 517 cases diagnosed with ADHD. Comprehensive details of the study protocol, epilepsy occurrence and characteristics, as well as EEG-epi-ab have previously been published [12, 29]. The EEGs of children diagnosed with ADHD were categorised based on the presence or absence of EEG-ab into abnormal or normal EEG (EEG-n). Patients with EEG-ab were further categorised into EEG-non-epi-ab group and EEG-epi-ab group. The EEG-epi-ab group was then subdivided into cases with and without EEG-non-epi-ab. EEG-epi-ab were defined as spikes or spike-wave complexes, isolated or occurring serially (in runs) without evident clinical signs of epileptic seizures (SZ). SZ, epilepsy and epileptic syndromes were diagnosed in accordance with the International League Against Epilepsy classification system [30–32]. SZ frequency during the last year was classified as SZ free, 1–12 SZ per year and > 12 SZ per year. Antiepileptic treatment (AED treatment) was categorised as untreated, monotherapy or polytherapy. Drug resistant epilepsy was defined as failure of adequate trials of two tolerated, appropriately chosen and used AED scheduled (whether as monotherapies or in combination) to achieve sustained seizure freedom. Baseline measure outcomes included the occurrence of EEG-ab, ADHD inattentive subtype (ADHD-I), previous epilepsy, SZ frequency, the use of antiepileptic drugs (AEDs) and the use of methylphenidate (MPH) in children with and without EEG-ab. MPH administration followed the Norwegian Guidelines [33], with dosage ranging from 0.5 to 1.2 mg per kilo, either three times daily (short-acting MPH) or once per day (slow-acting MPH). During titration with MPH the use of AEDs was stable. The response to MPH was assessed after 4–6 weeks of treatment and considered positive if a significant reduction in ADHD symptom scores, as assessed with ADHD IV rating scale (ADHD-RS-IV total score ≤ 18), was observed [34], alongside parental and teachers observations. In a 3-year follow-up, all 39 patients with EEG-epi-ab were compared with 39 randomly selected age- and gender-matched ADHD controls without EEG-epi-ab.

Measure outcomes included the presence of EEG-ab on the last control EEG, maintenance on MPH, the use of AEDs, and the safety of MPH use (SZ risk) in cases with previous epilepsy and in cases with EEG-epi-ab with and without EEG-non-epi-ab. At least one control EEG was performed in cases with EEG-epi-ab. Maintenance on MPH was assessed based on participant and parental reports.

### Statistical analyses

Statistical analysed involved comparing continuous demographic and clinical variables in subjects with and without EEG-ab. Student’s t-test was used for continuous and symmetrically distributed data, while Mann-Whitney tests were applied for continuous and skewed data. Proportions were compared using Chi-squared test or Fischer’s exact test. Methods for matched samples (paired-samples t-tests, McNamara test) were also employed. However, the application of methods for matched samples did not alter the conclusion. A p-value of < 0.05 was considered statistically significant.

### Approval

The study was approved by the Norwegian Data Inspectorate and the Regional Committee for Medical Research Ethics Western Norway (nr.010.07). The study was performed in accordance with ethical standards of the Declaration of Helsinki. Written informed consent was obtained from parents.

## Results

### Demographic and clinical characteristics of 517 children diagnosed with ADHD at baseline

Table 1 presents the demographic and clinical characteristics of the 517 children diagnosed with ADHD at baseline. Table 2 outlines the clinical features of children diagnosed with ADHD and epilepsy, while Table 3 details the characteristics of 39 children with EEG-epi-ab compared to those with and without EEG-non-epi-ab.

**Table 1:** Sociodemographic and clinical variables at baseline

Variables	*N* = 517	EEG normal *n* = 244, (47.2%)	EEG- ab *n* = 273 (52.8%)	EEG non-epi-ab *n* = 264 (51.1%)	EEG-epi-ab *n* = 39 (7.5%)	EEG without EEG-epi-ab *N* = 478 (92.5%)
Age, mean	9.4 + 2.5	9.5 + 2.5	9,3 + 2.4	9,2 + 2.3	9.7 + 2.7	9.3 ± 2.4
Gender (m)	82%	86.1%	78.3%	82.8%	71.8%	83.1%
ADHD-I (n, %)	68 13.2%	27 11.1%	41 15%	39 14.9%	16 41%	50 10.5%
Epilepsy SZ, last year	14 3	1 (0.4%) 0	13 (4.8%) 3	9 (3.4%) 3	12 (30.8) 3	2 (4.2%) 0
MPH, at baseline	481 (93%)	225 (92.2%)	256 (93.8%)	246 (93.2%)	36 (92.3%)	443 (92.7%)
MPH, positive response	388 (80.7%)	178 (79.1%)	210 (82%)	204 (82.9%)	30 83.3	354 (79.9%)

ADHD-I = ADHD predominantly inattentive type; EEG-ab = EEG abnormalities; EEG-epi-ab = EEG epileptiform abnormalities; EEG-non-epi-ab = EEG non-epileptiform abnormalities; MPH = methylphenidate; SZ = epileptic seizures

**Table 2:** Clinical characteristics of children with ADHD with and without epilepsy

	Total (*N* = 517)	With epilepsy (*N* = 14)	Without epilepsy (*N* = 503)	*p*-value
Age, Mean + SD at diagnosis	9.4 (2.5)	8.2 (2.3)	9.4 (2.5)	0.07
Gender, male (%)	424 (82%)	10 (71.4)	490 (82.6%)	0,5
EEG-ab	273 (52.8)	13 (92.9%)	260 (51.7%)	0.02*
EEG-epi-ab	39 (7.5%)	12 (85.7%)	27 (5.4%)	0.001*
EEG-non-epi-ab	264 (51.1%)	10 (71.4%)	254 (50.5%)	0.1
MPH initial use	481 (93%)	14 (100%)	467 (92.8%)	0.3
MPH positive response	388 (80.7%)	12 (85.7%)	376 (74.8%)	0.35

EEG-epi-ab = EEG epileptiform abnormalities; EEG-non-epi-ab = EEG non-epileptiform abnormalities (slowing and/or irregularity of the background rhythm); MPH = methylphenidate; SZ = epileptic seizures

All significant differences are between the group with epilepsy and the group without epilepsy

**Table 3:** Subdivision of the 39 children with EEG-epi-ab: those who also had non-epi-ab, and those who did not have non-epi-ab

Variables	EEG-epi-ab *n* = 39	EEG- non-epi-ab 30 (76.9%)	EEG without non-epi-ab 9 (23.1)
Age, mean *±* SD	9.7 + 2.7	9.6 + 2.8	10 + 2–6
Gender (m)	71.8%	70%	77.8%
ADHD-I (n, %)	16 (41%)	14 (46.7%)	2 (22.2%)
Epilepsy SZ, last year	12 (30.8) 3	9 0	3 0
MPH, at baseline	36 (92.3%)	28 (93.3%)	8 (88.9%)
MPH, positive response	30 (83.3%)	24 (85.7%)	6 (75%)

ADHD-I = ADHD predominantly inattentive type; EEG-epi-ab = EEG epileptiform abnormalities; EEG-non-epi-ab = EEG non epileptiform abnormalities (slowing and/or irregularity of the background rhythm); MPH = methylphenidate; SZ = epileptic seizures

EEG-ab were identified in 273 (52.8%) cases, a significantly higher prevalence than anticipated in healthy children (10–30% [35–38] and the assumed general population rate of 10% [39]. The EEG-ab group exhibited a higher proportion of girls (21.7% vs. 13.9%). There were no statistically significant differences between the groups regarding age, gender, ADHD-I type or initial use of MPH. While the percentage difference in ADHD-I among cases with and without EEG-ab was not significant, the ADHD-I type was notably more significantly prevalent in the EEG-epi-ab group (41% vs. 14.9%) compared to children with EEG-non-epi ab 14.9% and those with a normal EEG 11.1%.

EEG-non-epi-ab characterised by slowing and/or background irregularity were significantly more prevalent in cases with EEG-epi-ab compared to those patients with EEG-epi-ab who did not have EEG non-epi-ab (30/39, (76.9%) vs. 9/39, (23.1%). Additionally, EEG-non-epi-ab was identified in 234/478 (49%) cases without EEG epi-ab. Among the 14 children with a history of epilepsy, 13 (92.9%) exhibited EEG-ab, 12 (85.7%) had EEG-epi-ab and 10 (71.4%) displayed EEG-non-epi-ab. In contrast, among the 503 children without prior epilepsy, 263 (52.3%) had EEG-ab, 27 (5.4%) had EEG-epi-ab and 254 (50.5%) had EEG-non-epi-ab. The initial positive response to MPH was comparable in cases with and without EEG-ab (82% vs. 79%). No statistical difference was observed between the groups in terms of to age, gender, ADHD-I type or initial use of MPH. Of the 39 patients with EEG-epi-ab, 36 were initially treated with MPH. All children with epilepsy received MPH, and AEDs, with 11 on monotherapy and 3 on polytherapy. All children with epilepsy had recent SZ (last 5 years). In the past year, 11 patients were seizure-free, two had 1–12 SZ and one had more than 12 SZ. Notably, 64.3% (9/14) of children with epilepsy had localisation-related epilepsy while 35.7% had generalised epilepsy.

### Three-year follow-up of 39 cases with and 39 matched controls without EEG-epi-ab

During follow-up period, additional assessments were conducted for cases with EEG-epi-ab, including a control routine awake EEG for all 39 cases with EEG-epi-ab at baseline, a sleep EEG for 15 patients and long-term video-EEG monitoring for 6 cases. Notably, one case exhibited continuous spike waves during slow sleep on EEG recording, prompting treatment with Levetiracetam. However, a significant reduction in ADHD symptoms was not observed. The initial treatment involved stimulants, followed by a combination of Atomoxetine and Leveritacetam. No control EEG was performed for the 39 cases in the control ADHD group without EEG-epi-ab at baseline because of limited resource. EEG assessment was not used regularly in follow up of ADHD patients, only if occurrence of SZ was suspected.

#### Disappearance or persistence of EEG-ab on control EEG

Upon the 3-year follow-up of the 39 cases with EEG-epi-ab at baseline assessment, 11/39 (28.2%) still exhibited EEG-epi-ab on the last control EEG. Among the 12 patients with previous epilepsy, 4 displayed EEG with focal epileptiform abnormalities. In contrast, among the 30 cases with baseline EEG-non-epi-ab, 20/30 (66.7%) continued to show EEG-non-epi-ab on the control EEG. The disappearance rate of EEG-epi-ab was notably higher than that of EEG-non-epi-ab (71.8% vs. 33.3%).

#### EEG-ab and risk for SZ

At baseline, all 14 children with prior epilepsy received AEDs. Three years later, only 7 children (50%) continued to receive AEDs, with 5 on monotherapy and 2 on polytherapy. Among the 39 children with EEG-epi-ab and 14 with epilepsy, only 3 children with previous difficult-to-treat epilepsy (drug resistant epilepsy) had experienced SZs during the 3-year follow-up, with no change in SZ frequency. One of them achieved SZ freedom during the last year follow-up. The use of AEDs and MPH in 14 ADHD children with epilepsy are presented in the Table 4.

**Table 4:** The use of AEDs and MPH in 14 ADHD children with epilepsy

Demographics	14 ADHD patients with epilepsy
Baseline at epilepsy diagnosis, age, mean	6.4 y
AEDs, n (%) Monotherapy, Polytherapy	14 (100%) 12 (VPA 4, CBZ 3, OXC 2, LTG 1, LEV 1, SUL 1) 2 (LTG + TPM 1, TPM + ESM 1)
Baseline at ADHD diagnosis, age, mean	8.2 y
Gender, male/N, (%)	10 (71.4%)
MPH initial use n (%)	14 (100%)
MPH positive response N (%)	12 (85.7%)
SZ occurrence	3 (in cases with drug resistant epilepsy)
AEDs Monotherapy, Polytherapy	12 10 (VPA 3, CBZ 2, OXC 2, LTG 1, LEV 1, SUL 1) 2 (LTG + TPM 1, TPM + ESM)
3 years follow-up	
MPH n/N (%)	7/12 (58.3%)
Sz occurrence	2 (in cases with drug resistant epilepsy)
AEDs Monotherapy Polytherapy	7/14 (50%) 5 (VPA 1, CBZ 1, OXC, 1 LTG 1, LEV 1 2 (LTG + TPM 1, TPM + ESM 1)

AEDs, antiepileptic drugs; ESM, ethosuximide; LEV, levetiracetam; LTG, lamotrigine; MPH, methylphenidate; OXC, oxcarbazepine; SUL, sulthiame; SZ, epileptic seizures, TPM, topiramate; VPA, valproate

#### Maintenance on MPH three years later

In general, there were no significant differences between the groups with and without EEG-epi-ab in the utilization of MPH at the three-year follow-up. The rates of maintenance on MPH were comparable for cases with and without EEG epi-ab (86.7% vs. 81.8%). Additionally, we conducted an analysis of maintenance on MPH in cases with EEG-epi-ab comparing those with EEG-non-epi-ab. We observed a higher percentage of individuals maintaining MPH treatment in cases with EEG-epi-ab associated with EEG non-epi-ab compared to cases with EEG-epi-ab without EEG-non-epi-ab (91.7% vs. 66.7%, with 22 out of 24 and 4 out of 6 individuals, respectively). (Clinical characteristics of 39 patients with EEG-epi-ab and 39 ADHD controls without EEG-epi-ab are presented in the Table 5.

**Table 5:** 39 cases with and 39 controls without EEG-epi-ab at 3 years follow-up

	39 cases with EEG-epi-ab			39 controls without EEG-epi-ab			P
		With non-epi-ab	Without non-epi-ab		With non-epi-ab	Without non-epi-ab	
N, (%)	39 (100%)	30 (76.9%)	9 (23.1%)	39	19 (48.7%)	20 (51.3%)	0.1
Age, mean + SD	9.7 *±* 2.7	9.6 *±* 2.8	10 *±* 2.6	9.7 *±* 2.7	10.*±*2.6	9.4 *±* 2.7	
Gender, female (%)	11 (28%)	9 (30%)	2 (22.2%)	11 (28%)	4 (21.1%)	7 (35%)	
ADHD-I (%)	16 (41%)	14	2	6 (15.4%)	3	3	*P* < 0.01*
Previous epilepsy	12	8	4	0	0	0	*P* < 0.001
MPH, at baseline (%)	36 (92.3%)	28	8	37 (94.9%)	17	20	0.65
MPH, positive response (%)	30 (83.3%)	24	6	33 (89,2%)	15	18	0.47
MPH, maintenance at 3 years (%)	26 (86.7%)	22 (91.7%)	4 (66.7%)	27 (81.8%)	12 (70.6%)	15 (75%)	0.49 0.11
Control EEG	39	20	19				
EEG-epi-ab	11 (28.2%)	7 (35%)	4 (21.1%)				
SZ occurrence (any SZ within the past 3 years)	3	3	0	0	0	0	

ADHD-I, ADHD predominantly inattentive type; EEG-epi-ab, EEG epileptiform abnormalities; EEG-non-epi-ab, EEG non-epileptiform abnormalities; MPH, methylphenidate; SZ, epileptic seizures

The significant difference is between the group with EEG epi-ab and the group without EEG-epi-ab

## Discussion

In this study, our objective was to explore the prevalence of EEG-ab among children diagnosed with ADHD during their baseline assessments, conducted using standard EEG protocols. We sought to understand the impact of EEG abnormalities on ADHD-I (it concerns 68 out of 517, and that the others were diagnosed with ADHD combined type), as well as the initial use of medications such as methylphenidate (MPH) and antiepileptic drugs (AEDs).

Additionally, our analysis extended to the persistence of EEG-ab observed in control EEG assessments, the risk of SZ, and the continued use of MPH in individuals with and without EEG-epi-ab 3 years post-diagnosis. This investigation aimed to determine whether incorportating a standard EEG examination could enhance our comprehension of ADHD, a disorder characterised by etiological heterogeneity.

### ADHD and occurrence of EEG-ab

The investigation of EEG-non-epi-ab has not been systematically explored in research studies. One possible explanation is that EEG is not routinely conducted during ADHD assessments and is typically considered only if epilepsy is suspected [40, 41]. We hypothesize that EEG-ab (especially EEG-non-epi-ab) occur more often in children with ADHD, compare with healthy controls, and may be associated with ADHD, but also with epilepsy. We wanted to analyse the association between EEG-ab and ADHD-I, the use of AEDs, and MPH at baseline ADHD assessment, and at three years follow-up.

In our study, 273 (52.8%) of the unselected sample of 517 children with ADHD exhibited EEG-ab, encompassing both EEG-non-epi-ab (51.1%) and EEG-epi-ab (7.5%). These percentages significantly surpass the prevalence of EEG-ab found in healthy children [35, 42–44]. The incidence of EEG-epi-ab in healthy children is reported to be 2,4–5% [35, 43, 45, 46]. As for EEG-non-epi-ab in healthy children, the incidence is 10–30% in the age range of 1–15 years [42], 4.9% in adolescents aged 16–21 years [38] and 10% in the general population [39].

The prevalence experiences a significant decline in young adult life [44]. Some background slowing, primarily in theta and delta, is often found in children during wakefulness, but excessive slowing is considered as pathological. A study by Kanazava et al. (2014) reported abnormal EEG findings in 48.3% (70/145) of newly diagnosed ADHD patients with an age range between 5 years, 9 months and 19 years, 9 months, and a mean age of 11 years, 4 months [13]. Our study revealed that children with EEG-epi-ab more frequently exhibited EEG-non-epi-ab compared to children with EEG-epi-ab alone (76.9% vs. 23.1%). The association between EEG-epi-ab, epilepsy occurrence and ADHD has been documented in numerous publications [4–9, 47–49]. It is anticipated that the occurrence of epilepsy is significantly higher in children with EEG-epi-ab than in those with a normal EEG, a finding corroborated in this study. Additionally, we observed that epilepsy occurs more frequently in children with EEG-non-epi-ab than those without (3.4% vs. 0.4%). In epilepsy patients interictal slowing in the region of interictal epileptiform discharges usually reflects the same underlying process and risk for SZ. In contrast, non-specific slowing is often associated with delayed maturation of EEG, and in our study occur in 51.1% of children with ADHD at baseline assessment.

### MPH use and initial response to MPH

The initial use and response to MPH in children diagnosed with ADHD, both with and without EEG-ab, exhibited similar patterns (Table 1). All children diagnosed with epilepsy were administered AEDs and the usage of AEDs did not demonstrate an association with diminished usage of MPH or a reduced positive response to MPH at baseline. Previous research has indicated that MPH treatment leads to clinically significant improvements in ADHD symptoms in 60–75% of patients [50]. Our findings suggest that the presence of EEG-ab and/or epilepsy at the time of ADHD assessment did not exert an influence on the initial utilisation or the positive response to MPH.

### Three-year follow-up (disappearance of EEG-ab, risk of SZ and maintenance on MPH)

During the follow-up period, we observed EEG normalisation and the disappearance of both EEG-epi-ab and EEG-non-epi-ab in the majority of patients. Among the 27 children in our study who initially presented with only EEG-epi-ab without epilepsy, 20 (74.1%) children exhibited the resolution of EEG-epi-ab upon subsequent EEG assessments. This phenomenon of EEG normalisation during follow-up and the resolution of EEG-epi-ab in healthy children has previously been documented by Cavazzuti and coworkers in 1980 [45]. Their research, spanning an 8–9-year timeframe, revealed the spontaneous disappearance of EEG abnormalities, typically occurring within the school-age period or, at the latest, during adolescence. Among the 131 cases studied, only 7 developed generalised tonic-clonic SZ, which responded well to AEDs. The researchers concluded that epileptiform EEG patterns are frequently observed in found in children during school years and, in the majority of instances, lack a clinical association with epilepsy. Interestingly, half of the patients with EEG abnormalities in Cavazzuti et al.‘s study also exhibited behaviour problems and/or slight psychomotor ability disturbances [45]. In our baseline assessment, EEG-epi-ab was identified in 85.7%, (12/14) of cases with a history of epilepsy. Of these 12 children with epilepsy and EEG-epi-ab, 8 (66.7%) children displayed EEG patterns without EEG-epi-ab in subsequent control EEG assessments.

One study conducted by Hesdorfer et al. (2004) [51], proposed a potential link between ADHD and the development of epilepsy. However, our investigation did not reveal a heightened susceptibility to new SZ among ADHD-diagnosed patients without pre-existing, challenging-to-treat epilepsy during the three-year follow-up. Although we cannot rule out the possibility that some of these individuals might develop SZ later in life, our findings contrast with those of Hemmer et al. (2001), who examined the SZ risk in non-epileptic ADHD patients using stimulants [49]. The patients were diagnosed between 1993 and 1998, and in 1999 were followed up with either an office visit or telephone contact. In their study, 10%, 3 out of 30 patients with EEG-epi-ab experienced SZ during follow-up. They suggested that EEG-epi-ab in neurologically normal children with ADHD predicted a considerable risk of to eventual SZ occurrence. In our study involving 27 cases with EEG-epi-ab but no prior history of epilepsy, none developed SZ during follow-up. Furthermore, the frequency of SZ in cases with difficult-to-treat epilepsy remained unchanged from baseline, and none of 47 children with EEG-non-epi-ab without difficult-to-treat epilepsy developed SZ. Our results are concordant with several studies examining the use of MPH in children with ADHD and epilepsy or EEG-ab [50, 52–57], and with larger database/registry studies demonstrating no increase in SZ with ADHD medication use [58, 59]. Notably, our study demonstrated the safety of MPH use in children with ADHD despite the presence of EEG-ab, EEG-epi-ab and epilepsy, during the three-year follow-up, although there are too few subjects with SZ to draw any definite conclusions.

Patients with ADHD treated with MPH often have decreased compliance over time and adherence to treatment at follow up can be suboptimal. Treatment compliance is influenced by various factors such as medication type and patient age [60]. Among children and adolescents treated with long-acting MPH, adherence ranged from 49% in adolescents to 59% in children [61]. Discontinuation rates for stimulants varied from 19.1% for long-acting stimulant users of all ages to 99% for slow-acting MPH paediatric (6–12 years old) users [62]. Surprisingly, our findings indicate similar maintenance on MPH three years later in children with EEG-epi-ab compared to a control group of children without EEG-epi-ab. When comparing groups of children with EEG-epi-ab with and without EEG-non-epi-ab, a higher rate was observed in the group with both EEG-epi-ab and EEG-non-epi-ab. This finding is based on a small sample of ADHD children, but one can speculate that children with both EEG-epi-ab and EEG-non-epi ab more often have neuropsychiatric dysfunctions, which contribute to the ADHD symptoms.

Clark et al. (2019) explored EEG changes from childhood to adulthood. The study involved the assessment of twenty-five male adults diagnosed with ADHD, initially evaluated between the ages of 8–12 years and later re-evaluated during adulthood. In comparing adult ADHD individuals with control subjects, the findings generally suggest a decrease in the extent of EEG abnormalities observed in these individuals during childhood. However, noteworthy distinctions persist [62]. One could hypothesise that the enduring presence of EEG-non-epi-ab may be associated with sustained use of MPH in children initially exhibiting EEG-epi-ab. Our EEG recordings were conducted during wakefulness without sleep deprivation utilising the same centre (for more description see Socanski et al. 2010) ensuring uniform conditions for all patients. Due to resource constraints, additional EEG assessments were not performed on 39 cases from the control group without EEG-epi-ab. Exploring whether an elevated rate of MPH maintenance is also evident in ADHD patients with previously recorded EEG-non-epi-ab but without EEG-epi-ab could be of considerable interest.

### Strengths, limitations, and future research

#### Strengths of the study

Our study boasts notable strengths, including a sizable, highly representative, and unbiased cohort. All children suspected of having ADHD were referred to the centre, and patients were systematically assessed in consecutive order. Notably, there were no alternative treatment facilities in the region, making our study the most extensive study where patients were prospectively collected and analysed at a single regional centre.

#### Limitations of the study

A limitation in our study is the relatively low number of cases with epilepsy (*N* = 14) and EEG-epi-ab (*n* = 39), along with the absence of a prospective and randomised controlled trial during the 3-year follow-up. Resource constraints prevented a follow-up assessment of all cases.

#### Future research

The groups with EEG-epi-ab and the control group without EEG-epi-ab were followed up for three years. Future investigations might consider reassessing these groups, perhaps 10 years later. While our study did not establish a connection between EEG-ab and/or EEG-epi-ab at baseline and the risk of new SZs in children without a previous history of difficult-to-treat epilepsy, this conclusion was drawn from a relatively small sample (*n* = 27). Therefore, our findings warrant confirmation in a larger study. As per DSM 5, ADHD symptoms and autistic features may coexist in the same child, and comorbid diagnoses are now recognised. EEG-epi-ab and epilepsy occur more frequently in children with autism (up to 30%) compared to their healthy counterparts. However, it remains a subject of investigation whether EEG-epi-ab exerts an influence on the emergence of ADHD symptoms, cognitive, behavioural disturbances as well as autistic symptoms. The likelihood of encountering EEG-epi-ab and/or epilepsy is anticipated to be higher in cases where both ADHD and autism coexist, demanding careful consideration and interpretation during diagnostic assessments.

## Conclusions

EEG-ab are more frequently observed in children with ADHD in comparison to both the general paediatric population and healthy children. Among children with ADHD there were no notable distinctions in terms of age, gender, the occurrence of ADHD-I, and initial use of MPH whether they exhibited EEG abnormalities or not. The presence of EEG- ab either with or without accompanying EEG-epi-ab, did not correlate with an elevated risk of developing seizures (SZ) during the three-year follow-up period, even when taking into account the use of MPH. It is noteworthy that the majority of children exhibited a normalisation of EEG-ab upon subsequent EEG assessments. Additionally, the groups characterised by the presence or absence of EEG-epi-ab showed similar patterns of MPH usage, including initial use, positive response to MPH, and continued maintenance on MPH.

## Data Availability

No datasets were generated or analysed during the current study.
